# Neonatal Outcome of Mothers With COVID-19 in King Salman Armed Forces Hospital, Tabuk, Kingdom of Saudi Arabia

**DOI:** 10.7759/cureus.45257

**Published:** 2023-09-14

**Authors:** Malak Mohamed Eltayeb, Rofayda Mansour Ahmed Mohamad, Iftiraj Sulaiman Alhawiti, Ghalib Mohammed Alsulami, Samir Salah Eldin Mohamed Buraei, Sakeina Saleem Haroon Mohammed, Hosam Hadi Awaji

**Affiliations:** 1 Pediatrics Department, King Salman Armed Forces Hospital, Tabuk, SAU; 2 Preventive Medicine Department, King Salman Armed Forces Hospital, Tabuk, SAU; 3 Family Medicine Department, King Salman Armed Forces Hospital, Tabuk, SAU

**Keywords:** cesarean section, pregnancy outcome, mothers, fetal outcome, covid-19

## Abstract

Objectives: This study was conducted to assess the neonatal outcome of mothers with COVID-19 in King Salman Armed Forces Hospital, Tabuk, Saudi Arabia.

Methods: This was a hospital record-based, retrospective cohort study. The case group included neonates born to mothers who were positive for the COVID-19 virus during pregnancy, whereas the control group included neonates born to mothers who were not infected with the COVID-19 virus during pregnancy. The data were collected from the records and were analyzed using the Statistical Package for the Social Sciences software (IBM Corp., Armonk, NY, USA).

Results: This study covered the hospital records of 342 women (114 cases and 228 control). The rates of cesarean sections and small for gestational age were significantly higher among the cases compared to the controls (71.1% versus 43.4%, p < 0.001 and 24.6% versus 11.8%, p = 0.003; respectively). The mean birth weight was significantly lower among the cases group (3.0 ± 0.6 versus 3.3 ± 0.6 kg, p = 0.022). Only the case group reported the occurrence of neonatal COVID-19 infection (7.9%, p < 0.001). The study reported only a single case of intrauterine fetal death and one stillbirth in the cases group, but no neonatal deaths (p > 0.05).

Conclusions: Maternal COVID-19 may be associated with undesirable neonatal outcomes. There is a possibility of vertical transmission of COVID-19 from the mother to the neonate, but this cannot be confirmed.

## Introduction

The coronavirus disease 2019 (COVID-19) has spread rapidly across the world, causing a pandemic that affected over 700 million confirmed cases and resulted in fatalities amounting to six million since the pandemic outbreak till August 2023 [1].

Pregnant women are at a higher risk of contracting viral infections, including COVID-19 infection, as pregnancy induces a state of immune tolerance [2]. Viral infections expose pregnant women to a higher risk of severe complications [3].

Concerns have been raised regarding the impact of COVID-19 infection in pregnant women on their neonates. Several studies reported maternal COVID-19 infection during pregnancy was associated with neonatal morbidity and mortality [4-12].

The pathogenesis of COVID-19's negative effect on fetuses and newborns is attributed to the increased coagulability state in mothers infected with COVID-19, leading to thrombotic changes in the placental vasculature with placental malperfusion and subsequent chronic fetal hypoxia [13,14], which can result in fetal distress, preterm labor, and stillbirth [8].

According to the best knowledge of the researchers, there are no published reports in Saudi Arabia on the neonatal outcomes of COVID-19-positive pregnant women. Therefore, this study attempted to assess the neonatal outcome of mothers with COVID-19 who gave birth at King Salman Armed Forces Hospital, Tabuk, Saudi Arabia, between January 2021 and January 2022.

## Materials and methods

Ethical considerations

The proposal for this study was approved by the Ethics Committee of King Salman Armed Forces Hospital, Tabuk, Saudi Arabia (Approval number: KSAFH-REC-2022-451). The data confidentiality was assured by keeping the datasheet anonymous after assigning a specific code for each patient that is known only by the investigators.

Study design and setting

This hospital record-based, retrospective cohort study was conducted at King Salman Armed Forces Hospital, Tabuk, Saudi Arabia.

Eligibility criteria

Eligible records were those of mothers who gave birth at King Salman Armed Forces Hospital, Tabuk, Saudi Arabia between January 2021 and January 2022. Only records with complete data of interest were included. Hospital records were excluded if relevant data were missing and if the pregnancy ended before 34 weeks of gestation.

Data collection

The total coverage method was used by recruiting all the neonates born to mothers who were PCR-positive for COVID-19 infection during the study period (their number is 120 according to records), in addition to the other group: the group of newborns to mothers who were not positive for the virus (240 participants), with a ratio of 1:2, with group matching to control any potential confounders. Testing with PCR was conducted only for mothers who showed manifestations suggestive of COVID-19.

The data of the study were collected by the research team from the patient’s hospital records using a comprehensive, structured, closed-ended checklist. Eligible records were divided into two groups: the case and control groups. The case group included neonates (34 weeks of gestational age or more) born to mothers who were positive for the COVID-19 virus during the last two weeks of pregnancy. Meanwhile, the control group included neonates born in the same place and time as mothers who were not infected with the COVID-19 virus during pregnancy.

The hospital’s policy for the management of COVID-19-infected mothers and their newborns was based upon the statement issued by the Saudi Ministry of Health. COVID-19-positive mothers were isolated and treated according to the disease manifestations and severity. The newborns of positive mothers are also isolated, and PCR analysis to diagnose infection with COVID-19 was conducted twice.

Statistical analysis

Data were entered, cleaned, and analyzed using Statistical Package for Social Sciences (IBM SPSS Statistics) for Windows, version 28 (IBM Corp., Armonk, NY, USA). Descriptive statistics were presented in the form of frequencies for categorical variables and means with standard deviations for numerical variables. Bivariate analysis was performed to determine the difference in the neonatal outcomes between the two study groups regarding the characteristics of the participants and the perinatal outcomes. The comparisons were carried out using Pearson’s Chi-squared statistical test (for categorical variables) and unpaired t-test (for numerical variables). A P-value of less than 0.05 was considered statistically significant.

## Results

This study covered the hospital records of 342 women who were divided into two study groups according to their history of confirmed COVID-19 infection by PCR analysis in the last two weeks of their pregnancy. Out of the available records for 120 COVID-19-positive pregnant mothers within the last two weeks before delivery, 114 were included in the case group while six patients were excluded due to incomplete records or delivery before 34 weeks of gestation. The control group included 228 mothers who were not diagnosed with COVID-19 within the last two weeks of pregnancy.

All the studied neonates were delivered at or after the 34th week of gestation according to our inclusion criteria. The study showed that the rate of cesarean sections was significantly higher among the cases compared with controls (71.1% versus 43.4%, p < 0.001). The mean heart rate was significantly higher among cases compared with controls (153 ± 19.2/min versus 147.4 ± 8.3/min, p = 0.002). Furthermore, the occurrence of fetal distress (manifesting as tachypnea with increased breathing efforts and oxygen requirement) was significantly higher among the cases group compared with controls (13.2% versus 5.3%, p = 0.011). All neonates of COVID-19 mothers were isolated and observed, with PCR testing done for them two times. They were discharged after obtaining negative swabs. The mean duration of hospital stay was significantly longer among the cases group compared with controls (7.2 ± 7.9 days versus 2.0 ± 1.7, p < 0.001). Both the cord thyroid stimulating hormone and hemoglobin were significantly lower in the cases group compared with controls (p < 0.05 in all), but the findings were almost similar in both study groups concerning bilirubin, total white blood cells, and platelets (p > 0.05 in all) (Table 1).

**Table 1:** Comparison between the study groups regarding the neonatal characteristics and investigations COVID-19: coronavirus disease 2019; SD: standard deviation; TSH: thyroid-stimulating hormone; WBCs: white blood cell count; *significant

Variables			Case (N = 114)	Control (N = 228)	P-value
Delivery	Caesarian section	N (%)	81 (71.1%)	99 (43.4%)	< 0.001*
Neonatal characteristics	Birth weight (kg)	Mean ± SD	3.0 ± 0.6	3.3 ± 0.6	0.022*
	Birth length (cm)	Mean ± SD	49.6 ± 4.9	50.2 ± 2.5	0.385
	Small for gestational age	N (%)	28 (24.6%)	27 (11.8%)	0.003*
	Heart rate (beat/minute)	Mean ± SD	153 ± 19.2	147.4 ± 8.3	0.002*
	Fetal distress	N (%)	15 (13.2%)	12 (5.3%)	0.011*
	Hospital stay (days)	Mean ± SD	7.2 ± 7.9	2.0 ± 1.7	< 0.001*
Investigations	Cord TSH (mIU/mL)	Mean ± SD	5.4 ± 3.7	8.1 ± 7.7	0.003*
	Cord bilirubin	Mean ± SD	34.7 ± 9.7	34.8 ± 12.1	0.943
	Cord total WBCs (X10^3^/cm^3^)	Mean ± SD	15.0 ± 4.4	16.5 ± 6.4	0.360
	Hemoglobin (g/dL)	Mean ± SD	16.1 ± 2.2	17.7 ± 3.6	< 0.001*
	Platelets (X10^3^/cm^3^)	Mean ± SD	280.2 ± 62.4	270.0 ± 209.8	0.611

No significant differences were observed regarding the occurrence of other complications, including neonatal asphyxia, meconium aspiration, and congenital anomalies (as detected by ultrasound examination of the mothers during pregnancy). Similar findings were reported in Apgar scores between the two study groups within the first and the fifth minutes with no significant difference. Only the cases group reported the occurrence of COVID-19 infection (7.9%) in neonates, with a significant difference compared with the control group (p < 0.001 in both). The study reported only a single case of intrauterine fetal death and one case of stillbirth in the cases group, but no neonatal deaths (p > 0.05) (Table 2).

**Table 2:** Comparison between the study groups regarding the fetal/neonatal mortality COVID-19: coronavirus disease 2019; *significant

Neonatal infection/outcome		Maternal status for COVID-19 infection within the last two weeks of pregnancy				P-value
		Positive		Negative		
		Frequency	%	Frequency	%	
COVID-19 infection	Positive	9	7.9	0	0.0	< 0.001*
	Negative	105	92.1	228	100.0	
Pneumonia	Yes	0	0.0	0	0.0	-
	No	114	100.0	228	100.0	
Intrauterine fetal death	Yes	1	0.9	1	0.4	0.616
	No	113	99.1	227	99.6	
Stillbirth	Yes	1	0.9	0	0.0	0.159
	No	113	99.1	228	100.0	
Neonatal death	Yes	0	0.0	0	0.0	-
	No	114	100.0	228	100.0	

## Discussion

This case-control study was conducted to assess the neonatal outcome of mothers with COVID-19 in King Salman Armed Forces Hospital, Tabuk, Saudi Arabia, between January 2021 and January 2022. The hospital records of 342 women were revised and divided into two study groups according to their history of confirmed COVID-19 infection in the last two weeks of their pregnancy. The cases group consisted of 114 mothers with positive results for COVID-19 within the last two weeks before delivery, while the control group included 228 mothers who were not diagnosed with COVID-19 within the last two weeks of pregnancy.

The current study revealed that the cases group had significantly higher rate of cesarean sections compared to the control group (71.1% versus 43.4%, p < 0.001). These findings are in line with systematic reviews and meta-analyses which reported that the rate of cesarean section in COVID-19-positive mothers was 83.5% [15] and 86% [16]. Another meta-analysis showed that COVID-19-positive pregnant women had a higher likelihood of cesarean delivery (odds ratio (OR): 1.20; 95% confidence interval (CI): 1.10-1.30) [17]. The reasons for cesarean sections in the current study and previous studies were attributed to obstetric conditions, including repeated sections, fetal distress, and premature rupture of membranes [18,19] as well as the severe COVID-19 infection causing maternal respiratory insufficiency [20]. However, the mothers’ and obstetricians’ fear of vertical transmission may have contributed to the increased rate of cesarean section in pregnant women with COVID-19 infection [16,21]. However, a national cohort study reported a lower rate of cesarean delivery in the United Kingdom of 59%. These differences in reported rates of the route of delivery may be effectuated by the differences in dealing with COVID-19 in different countries and by the severity of outbreaks. The meta-analysis [15] and systematic review [16] included mainly studies from China and Iran that were conducted on a relatively smaller sample size compared to the national cohort study carried out in England [22].

As there is no clear evidence of vertical transmission of SARS-CoV-2 from the parturient mothers to the neonates, most clinical guidelines state that maternal infection with COVID-19 is not an indication for cesarean section. Therefore, the route and timing of delivery should be decided for each case, based on the disease severity, the presence of comorbidities, and the state of both pregnant women and fetuses. The American College of Obstetricians and Gynecologists (ACOG) even recommended postponing the delivery till having a negative test for COVID-19, if maternal and fetal conditions permit [23]. In the case of patients with severe illness (e.g., increased respiratory rate to 30 cycles/min or above, resting oxygen saturation of 93% or lower, respiratory failure necessitating ventilator, or shock with organ failure), termination of pregnancy may be indicated to lower the risk of maternal/fetal mortality [24].

This study found that the cases group had significantly higher heart rate (153 ± 19.2 versus 147.4 ± 8.3/min, p = 0.002) and rate of fetal distress (13.2% versus 5.3%, p = 0.011). These findings agree with the results of a meta-analysis by Jafari et al. [25] who reported fetal distress in 16% (95% CI: 7-32, p = 0.046). In addition, Jeong and Kim [17] found that the risk of fetal distress was significantly higher when mothers had COVID-19 infection (OR: 2.49; 95% CI: 1.54-4.03).

As regards the cord thyroid stimulating hormone and hemoglobin, both were significantly lower in the cases group compared with controls (p < 0.05). Thyroid hormones are involved in modifying inflammation and apoptosis; thus, they may affect placental development [26,27]. However, there is yet no evidence regarding the effect of thyroid dysfunction on the development of the placental barrier and vertical transmission of SARS-CoV-2. COVID-19 has been associated with anemia, but the exact mechanisms are not yet known. These may include inflammation and hemolysis caused by low-grade disseminated intravascular coagulation (Bergamaschi, 2021 #9018) or autoimmune hemolytic anemia (Nair, 2021 #9019).

There were no significant differences in the rates of other complications in the present study, including neonatal asphyxia, meconium aspiration, and congenital anomalies. In addition, the Apgar scores did not significantly differ between the two study groups within the first and the fifth minutes. These findings are in accordance with those of Papapanou et al. [6] in Greece, who reported that infected and non-infected fetuses had similar rates of abnormal Apgar scores and neonatal asphyxia. Also, Jafari et al. [25] reported a low rate (4%, 95% CI: 1.5-9, p = 0.8) of neonatal asphyxia in neonates of COVID-19-infected mothers. Moreover, Jafari et al. [25] reported mean one-minute and five-minute Apgar scores of 9 (95% CI: 8-10, p = 0.9) and 10 (95% CI: 9-10.7, p = 0.9), respectively, without significant differences between COVID-19 positive and negative participants.

As we stated earlier, the issue of vertical transmission of COVID-19 from the mother to the fetus is still controversial [4]. The uncertainty is partially attributed to the limitations of the available diagnostic tests [5] and the difficulty in excluding transmission to the newborn after delivery during breastfeeding and caring for the baby by the mother. We were unable to assess whether neonates were separated from the mothers directly after delivery and until testing for the COVID-19 virus. In the systematic review by Mirbeyk et al. [16], nasopharyngeal specimens were positive for COVID-19 in 11 only out of tested 219 neonates. A systematic review revised 47 studies to assess the rate of mother-to-fetus transmission of COVID-19 and found that transmission was confirmed in 0.3% only, probable in 0.5%, and possible in 1.8%, while transmission was unlikely in 80.3% and did not occur in 17% [28]. Further large-scale studies are required to assess the risk of vertical transmission of COVID-19 as - if the possibility of transmission exists and is high - this will have profound effects on the method of delivery and postnatal care.

As regards fetal and neonatal fatalities, the present study reported a single case of intrauterine fetal death and another case of stillbirth in the cases group. No neonatal deaths were reported. No statistically significant differences were detected between the studied groups. These findings are supported by the results of previous primary and secondary research. Zhu et al. [29] reported that only one neonate died. Moreover, Papapanou et al. [6] found comparable rates of stillbirth and neonatal deaths in infected and uninfected fetuses. In addition, the meta-analysis by Jafari et al. [25] showed a rate of neonatal deaths of 2.5% (95% CI: 1.5-6, p = 0.6), but the rate of stillbirth was 4% (95% CI: 1.5-10, p = 0.036) with a significant difference between COVID-19 infected and non-infected pregnant women.

The findings of the present study and the available body of evidence from the literature should be interpreted cautiously due to the difficulty of establishing a cause-effect relationship between maternal COVID-19 infection and neonatal outcomes. Several factors can affect fetal and neonatal outcomes, including the duration of infection and its timing during pregnancy, the severity of manifestations, and the status of the maternal immune system. In addition, COVID-19 infection places a burden on the mental health of pregnant women, subjecting them to psychosocial stress and depression. Psychosocial stress during pregnancy can negatively affect the mother and fetus, resulting in poor outcomes [16]. The limitations of the present study include the inability to obtain data regarding the severity of COVID-19 infection in the mothers, and thus we were not able to correlate the severity of infection with obstetric and neonatal outcomes. In addition, potential risk factors in both groups that may have affected the outcomes were not available, but the use of randomization methods in selecting the control group may partially reduce the effect of these confounders.

Although this study was conducted on a sufficient number of participants, it is a single-center study, which may affect the possibility of the generalization of the study’s results. In addition, we were unable to consider other factors that may contribute to pregnancy outcomes, such as the presence of maternal comorbidities, the severity of maternal infection with COVID-19, the occurrence of sepsis, and the separation between the newborn and the mother after birth. This is attributed to the study design (retrospective), which depended on the quality and quantity of data recorded in the patient’s hospital records.

## Conclusions

The results of this study revealed that COVID-19 may cause undesirable pregnancy outcomes, including low birth weight, fetal distress, and delivery by cesarean section. The results suggest a risk of neonatal infection with COVID-19, but environmental transmission after delivery could not be excluded. Thyroid dysfunction may play a role in increasing the risk of maternal infection and may affect the outcomes of pregnancy, but further research is necessary to elucidate this issue. The healthcare team carrying out the delivery of COVID-19-infected pregnant women should adhere to the necessary precautions for preventing COVID-19 transmission to the newborn or any of the team members. Separation of the newborn from the infected mother is indicated when the mother is severely or critically ill. There is a need for future studies to clear the causal link between these outcomes and COVID-19 infection. Future multi-center, large-scale prospective studies are recommended to assess all potential factors that may affect pregnancy outcomes and to give more broad and in-depth results.
